# Seroepidemiology Study of Porcine Epidemic Diarrhea Virus in Mexico by Indirect Enzyme-Linked Immunosorbent Assay Based on a Recombinant Fragment of N-Terminus Domain Spike Protein

**DOI:** 10.3390/microorganisms11071843

**Published:** 2023-07-20

**Authors:** Eduardo García-González, José Luis Cerriteño-Sánchez, Julieta Sandra Cuevas-Romero, José Bryan García-Cambrón, Francisco Jesus Castañeda-Montes, Francisco Villaseñor-Ortega

**Affiliations:** 1 Programa de Doctorado en Ciencias de la Ingeniería, Tecnológico Nacional de México en Celaya, Celaya 38010, Guanajuato, Mexico; 2 Centro Nacional de Investigación Disciplinaria en Salud Animal e Inocuidad, INIFAP, Mexico City 05110, Mexico; 3 Programa de Maestría en Biología Experimental, Universidad Autónoma Metropolitana Iztapalapa, Mexico City 09340, Mexico; 4 Programa de Estancias Posdoctorales por México para la Formación y Consolidación de las y los Investigadores por México, CONAHCYT, Mexico City 03940, Mexico; 5 Tecnológico Nacional de México en Celaya, Departamento de Ingeniería Bioquímica, Antonio García Cubas Pte #600 esq. Av. Tecnológico, Celaya 38010, Guanajuato, Mexico

**Keywords:** seroepidemiology, PEDV, iELISA, S protein

## Abstract

Porcine epidemic diarrhea (PED) is an intestinal disease caused by the porcine epidemic diarrhea virus (PEDV) and affects Mexico’s swine industry. Despite the disease initially being described in Mexico in 2013, there has been no research into the virus’s seroepidemiology carried out in Mexico. Thus, the goal of this study was to develop an indirect ELISA (iELISA) based on a recombinant N-terminal domain truncated spike (S) protein (*r*NTD-S) of PEDV to evaluate serum obtained from different pig-producing states in Mexico. A total of 1054 sera were collected from pig farms, slaughterhouses, and backyard production in the states of Aguascalientes, Guanajuato, Hidalgo, Jalisco, Morelos, Queretaro, Sinaloa, and Veracruz between 2019 and 2021. The *r*NTD-S protein was expressed in *E. coli* BL21 (DE3) cells. Negative and positive serum samples used in the iELISA were previously tested by Western blot. According to our findings, 61.66% of the serum samples (650/1054) were positive, with Jalisco having the highest percentage of positive samples, at a rate of 21.44% (226/1054). This is the first seroepidemiology study of PEDV carried out in Mexico, revealing that the virus is still circulating since the initial outbreak; furthermore, it provides an overview of PEDV’s spread and high level of persistence across the country’s key swine-producing states.

## 1. Introduction

Porcine epidemic diarrhea (PED) is a highly contagious infectious intestinal disease that is caused by the porcine epidemic diarrhea virus (PEDV). The disease is characterized by severe diarrhea, vomiting, and dehydration. PEDV infections can occur in pigs of all ages and are the most serious in piglets, with morbidity and mortality rates often reaching 100% and was first reported in Belgium in 1978 [1,2]. In Europe, the virus spread until the end of the 1990s [3,4,5] and is currently spreading throughout Southern Italy [6]. PEDV was found for the first time in the USA in 2013, and the causative agent had a high identity (99%) with Chinese strains isolated in 2012 [7,8] and PEDV has been considered endemic in USA ever since [9,10,11]. In Mexico, the National Health, Food Safety, and Food Quality Service (SENASICA) communicated to the World Organization for Animal Health (WOAH) in May 2014 the first PEDV positives cases, causing substantial economic losses to the swine industry. The molecular detection of PEDV was confirmed, and the nucleotide sequence analysis of Mexican samples indicated considerable similarities to viruses that had spread in Colorado, USA, in 2013 [12].

PEDV is an enveloped, single-stranded, positive-sense RNA virus [13]. The PEDV genome is approximately 28 kb in length. The genome encodes four structural proteins: glycosylated spike (S) protein (150–220 kDa); membrane (M) protein (20–30 kDa); envelope (E) protein (7 kDa); nucleocapsid (N) protein (58 kDa) [3,14,15]. The S protein is located on the virus envelope as a large surface projection of the virion and plays an important role in the attachment with the host cell receptor [16]. The S protein consists of two regions, S1 and S2. The S1 subunit is an extracellular domain that recognizes and binds to target cell receptors and is closely linked to the formation of neutralizing antibodies; thus, is considered the most antigenic protein of the virus [16,17]. Therefore, the gene and S protein are considered key pieces to understanding the genetic relationship and epidemiological states of PEDV field isolates, as well as in the development of biologics with potential use in vaccines [18]. The S1 region has been produced as a recombinant protein in a bacterial expression system for the development of an immunoenzymatic diagnostic system with high levels of sensitivity and specificity proposing them for serological evaluations [19]. The S1 subunit contains two domains, the N-terminal domain (NTD) and the C-terminal domain [19,20,21]. Moreover, a surface probability analysis of several samples of PED revealed a small number of differences in the NTD region of the S1 domain in Mexican strains from 2015 and 2016 compared to strains from previous years (2013–2014) with one isolate from 2013 exhibiting a more conserved NTD region. [22].

Currently, there is no comprehensive investigation into the seroepidemiology of the disease in Mexico. Estimating the number of pigs who have previously been infected with PEDV (seroepidemiology) enables an understanding of how the virus spreads in various locations, to retrospectively evaluate the performance of infectious disease models, and hence plan for further outbreaks. Therefore, in this study, an indirect ELISA (iELISA) based on a recombinant protein of the NTD-S fragment was developed to evaluate 1054 pig sera from a slaughterhouse and backyard production to provide information about seroepidemiology of the disease in Mexico.

## 2. Materials and Methods

**Figure 1:**
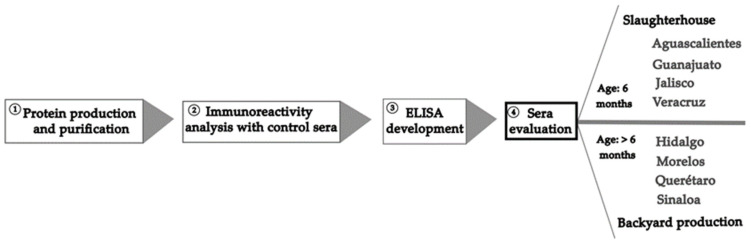
Schematic representation of the procedure followed in this study.

Figure 1 is a schematic representation of the procedure followed in this study to develop the recombinant protein of the NTD-S fragment and carry out the seroepidemiological study.

### 2.1. Production and Purification of Recombinant NTD-S Protein

*r*NTD-S was expressed and produced according to Lara-Romero et al., 2022 [22], the overproducing strain was donated by INIFAP CENID-SAI, Mexico City, and was named BL21-NTD-S. Briefly, 100 mL of LB medium was inoculated starting at 0.1 optical density units at 600 nm (OD_600 nm_), incubated at 37 °C, 250 rpm, and induced with IPTG 1.5 M (isopropyl β-d-1-thiogalactopyranoside) (IPTG; Merck KGaA, Darmstadt, Germany). Protein production was visualized by SDS-PAGE after staining with Coomassie Brilliant Blue G-250 (Bio-Rad, Hercules, CA, USA). The recombinant protein was confirmed by Western blotting using the 6X-His monoclonal antibody (Invitrogen, Rockford, IL, USA). The overproduction of recombinant proteins in *E. coli* was recovered from the inclusion bodies and purified according to Castañeda-Montes et al., 2023 [23]. Briefly, the inclusion bodies were solubilized in N-Lauroylsarcosine 5% and Tris-HCl 50 mM, pH 7.5 buffer. Later, the recombinant protein was purified using a Ni-NTA agarose column (5 mL) HisTrap^®^ Chelating High Performance (GE Healthcare, Chicago, IL, USA), and the pure *r*NTD-S was dialyzed against 300 volume of Tris-HCl 5 mM, pH 7.5 with 8 kDa regenerated cellulose membrane. Finally, the presence of purified *r*NTD-S protein was confirmed using SDS-PAGE and Western blotting, and protein concentration was determined via Bradford assay according to standard procedures.

### 2.2. Immunoreactivity of Control Swine Serum Samples to rNTD-S by Western Blot

A total of 30 negative serum samples from clinically healthy swine farms were obtained from a sera collection belonging to the Epizootiology laboratory, INIFAP CENID-SAI, Mexico City. Also, 30 positive sera samples were obtained from a farm experiencing PEDV infection and confirmed by RT-PCR. The positive sera had been collected from naturally infected pigs destined for supply at 6 months of age with a minimum of 3 months after infection. For Western blot analysis, 200 ng of purified *r*NTD-S protein was used to evaluate sera samples. Briefly, recombinant protein was transferred to a nitrocellulose membrane of 0.45 µm (Bio-Rad, CA, USA) and incubated overnight at 4 °C with blocking solution containing PBS-Tween buffer (137 mM NaCl, 2.7 mM KCl, 8 mM Na_2_HPO_4_, 2 mM KH_2_PO_4_, and 0.05% Tween-20) and 5% (*w*/*v*) skimmed milk powder. The membrane was then incubated with reference positive and negative sera (1:500 dilution in blocking buffer) for 2 h at room temperature on a shaker plate. Later, the membrane was washed three times with PBS-Tween buffer and incubated with HRP anti-pig IgG (Bethyl Laboratories Inc., Montgomery, TX, USA) (1:5000 dilution in blocking solution) at room temperature for 2 h. The protein bands were visualized with a developed solution (20 mL PBS; 0.0125 g 3,3′-diaminobenzidine (Sigma-Aldrich, St. Louis, MO, USA); and 20 µL hydrogen peroxide 30%). The Western blot results were classified by comparing the pixel intensity between a positive reference (hyperimmune serum sample) and other serum samples using the ImageJ program (Schneider C.A., Rasband). The following classification was determined: the sera with the highest degree recognition were designated as (****); the sera with moderate degree recognition were designated as (***); the sera with slight degree recognition were designated as (**); sera with light degree recognition were designated as (*); and, finally, the reference sera recognized as negative were designated as (−). The sera that had been evaluated as previously described were designated as references (negative and positive) for the development of iELISA.

### 2.3. Development of iELISA

Different concentrations of *r*NTD-S and serial dilutions of reference sera and anti-Pig-HRP were evaluated to obtain the optimal conditions for iELISA. Briefly, the assay was carried out on a MaxiSorpTM 96-well microplate (Nalge Nunc International Corp., Rochester, NY, USA) using different antigen concentrations ranging from 50 to 200 ng of purified protein per well in 50 mM carbonate–bicarbonate buffer (pH = 9.6) in a final volume of 100 µL, which were then absorbed overnight at 4 °C. The plate was then washed four times with PBS-Tween 0.1% and blocked with PBS-Tween with 5% skim milk for 1 h at 37 °C with moderate agitation. Subsequently, different dilutions of the positive and negative reference sera in a volume of 100 μL (1:50 to 1:250 diluted in blocked buffer) were absorbed into the microplate for 2 h at 37 °C with slight agitation. Then, the plate was washed four times with PBS-Tween, and different dilutions (ranging from 1:2000 to 1:15,000) of anti-Pig-HRP were placed in a volume of 100 μL for 1 h at 37 °C with slight agitation. Finally, the plate was washed with PBS-Tween, and 100 μL of 3,3′,5,5′-tetramethylbenzidine (TMB) (SeraCare, Milford, MA, USA) substrate solution was added to each well and incubated for different amounts of time in the dark. The reaction was stopped with 100 μL of 2 M sulfuric acid, and the optical density was measured at 450 nm (OD_450nm_). Finally, the results were analyzed using different graphics to determine the optimal conditions.

### 2.4. Determination of Specificity, Sensitivity, and Cut-Off Value

As described above, a collection of previously verified negative (*n* = 30) and positive (*n* = 30) sera assessed by Western blotting was used to establish the specificity, sensitivity, and cut-off value of the iELISA. Briefly, the positive and negative sera were analyzed using a 2 × 2 contingency table (Table 1).

The contingency chart shows the contingency for determining the specificity, sensitivity, and kappa index (Κ). The Κ index ranges from 0 to 1, where 0 indicates no agreement with the test and 1 indicates perfect agreement. The percentages of sensitivity and specificity were calculated with the following equations, respectively:

(1) $$\%\;\mathrm{sensitivity}=\left[\frac{a}{\;a+c\;}\right]\;*\;100$$

(2) $$\%\;\mathrm{specificity}=\left[\frac{d}{\;b+d\;}\right]*\;100$$

where a is the number of true positives; b is the number of false positives; c is the number of false negatives; and d is the number of true negatives. Moreover, the Κ was calculated by considering Western blot as the “gold standard” using the following equation:

(3) $$\;\kappa =\mathrm{Po}\;-\;\frac{\mathrm{Pe}}{1–\mathrm{Pe}}{,\;\mathrm{where}:\;P}_{o}=\frac{\;\left(\;a+d\;\right)}{N}{\;\mathrm{and}\;P}_{e}=\frac{\;r+t+s+u}{N^{2}}$$

where P_o_ is the proportion of agreements observed; P_e_ is the proportion of agreements expected in the hypothesis of independence; r is the sum value of a and b; s is the sum value of c and d; t is the sum value of a and c; u is the sum value of b and d; and N is the sum of r, s, t, and u. The interpretation of the kappa index value ranges from 1 (complete agreement) to 0 (agreement is equal to that expected by chance). Values of Κ > 0.81 mean nearly perfect agreement; 0.61–0.80 substantial agreement; 0.41–0.60 moderate agreement; 0.21–0.40 fair agreement; 0–0.20 slight agreement; and 0 poor agreement [24].

The cut-off value was defined as the average value of the optical density of the negative serum samples at OD_450nm_ plus 3 standard deviations (SD). Serum samples showing an OD greater than the cut-off value were considered PEDV-seropositive.

### 2.5. Evaluation of Serum Samples Obtained from Farms by iELISA

A total of 1054 (Table S1) sera samples from eight Mexican states were donated by local swine associations (Table 2): 774 pig sera from slaughterhouses in Aguascalientes, Guanajuato, Jalisco, and Veracruz (6 months old); and 280 pig sera from backyard production coming from Hidalgo, Morelos, Querétaro, and Sinaloa (>6 months old). It is important to mention that PED outbreaks possibly occurred in these states in 2019 and 2021. The sera were analyzed using the optimal conditions for the iELISA.

### 2.6. Data Analysis

A one-way ANOVA was used to statistically evaluate the results obtained from the sera analysis. Differences were considered statistically significant at *p* < 0.05, with a 95% confidence interval (* *p* < 0.05, ** *p* < 0.005, *** *p* < 0.0005). Plots and maps were constructed using SigmaPlot version 12.5 (Systat Software Inc., San Jose, CA, USA) and MapChart (https://www.mapchart.net/, accessed on 1 March 2023), respectively. All summarized data are presented as mean ± standard error of the mean (SEM).

## 3. Results

### 3.1. Expression and Purification of rNTD-S Protein

**Figure 2:**
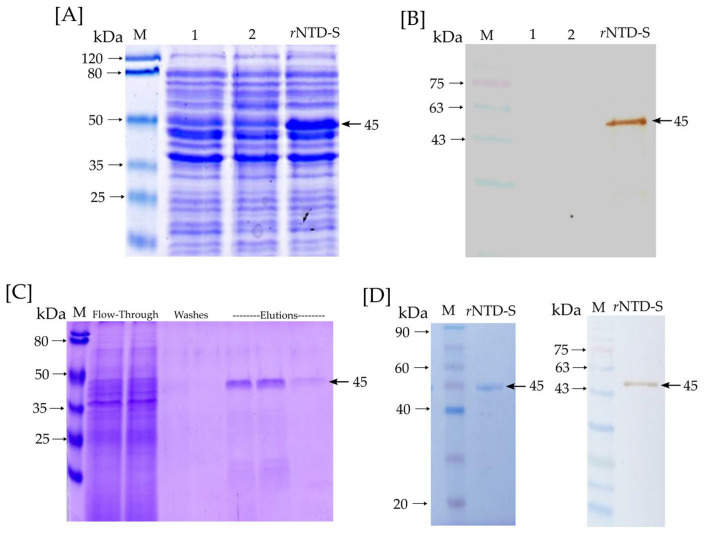
*r*NTD-S protein identification and purification: (**A**) SDS-PAGE and (**B**) Western blot—lines 1 and 2 correspond to lysate BL21 cells (negative control) and the *r*NTD-S line corresponds to lysate BL21-NTD-S cells (transformed cells); (**C**) Purifying process of *r*NTD-S with flow-through, washes, and elution phases; (**D**) SDS-PAGE and Western blot of purified and dialyzed *r*NTD-S protein. A specific signal of the *r*NTD-S protein at expected molecular weight (~45 kDa) is marked with a black arrow.

The *r*NTD-S protein was successfully expressed in the *E. coli* BL21(DE3) from an insoluble phase (inclusion body). Figure 2A,B show an SDS-PAGE and Western blot of the *r*NTD-S production. Lines 1 and 2 correspond to lysate BL21 cells (negative control), and the *r*NTD-S line corresponds to a lysate BL21-NTD-S (transformed cells). As can be observed, the *r*NTD-S line shows the production of the *r*NTD-S protein with ~45 kDa expected molecular weight. Moreover, Figure 2B shows a Western blot of a specific signal for the *r*NTD-S protein with ~45 kDa expected molecular weight. Figure 2C shows an SDS-PAGE with the purifying process and the elution phases show the presence of *r*NTD-S protein obtained by one step of affinity chromatography. On the other hand, Figure 2D shows an SDS-PAGE and Western blot of the dialyzed and purified *r*NTD-S protein with a specific signal of ~45 kDa expected molecular weight. Neither unspecific signals nor contamination was observed confirming the purified protein integrity. Finally, the purified *r*NTD-S was quantified via the Bradford assay, with an average yield of 56 µg per 100 mL of induced culture medium.

### 3.2. Analysis of Swine Serum Samples by Western Blot

**Figure 3:**
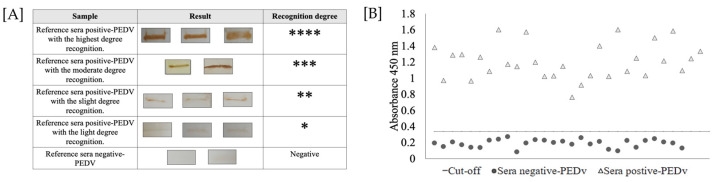
Immunoreactivity of purified *r*NTD-S protein. (**A**) Summary of Western blot analysis of the reference serum. Immunoreactivity was classified as follows: highest degree recognition (****); moderate degree recognition (***); slight degree recognition (**); light degree recognition (*); and negative sera (−). (**B**) Determination of the cut-off value using reference sera by iELISA. The dotted line represents the cut-off for iELISA and was calculated as 0.335, and 30 positive serum samples and 30 negative serum samples were evaluated using the developed iELISA. The sera above the dotted line were considered PEDV positive, and the sera below the dotted line were considered PEDV negative according to the cut-off value.

Immunoreactivity analysis of the *r*NTD-S protein was determined by Western blotting. As shown in Figure 3A, all positive swine sera showed specific recognition signals for the recombinant protein: three sera were strongly designated with four marks (****); five sera obtained a recognition degree of three marks (***); thirteen sera were designated with two marks (**); and nine sera obtained a recognition degree of one mark (*). In contrast, the analyzed reference sera were used as reference controls for iELISA development.

### 3.3. Standardization of iELISA with Purified rNTD-S Protein

The optimal iELISA condition was as follows: coated antigen dilution (*r*NTD-S protein) of 75 ng/well; 1:200 of serum sample dilution; 1:10,000 of secondary antibody anti-pig IgG-HRP. On the other hand, the positive and negative reference sera that had been previously analyzed were used to determine the percent sensitivity, specificity, and kappa index, and the results showed a sensitivity percentage of 96.77%, while the specificity percentage obtained was 100%, with concordance Κ = 0.966.

To determine the cut-off value of the iELISA, serum samples were evaluated using the optimal conditions described above. The average value OD_450nm_ of the reference sera was 0.188, and the standard deviation (SD) of the samples was 0.049. Thus, the cut-off value for the iELISA was 0.335. The highest value obtained for negative serum was 0.25 and the lowest was 0.08; moreover, the highest and the lowest values obtained for positive sera were 1.6 and 0.76, respectively. These values, both positive and negative, were different from the value obtained for the cut-off (Figure 3B). Unexpectedly, only one negative serum sample was recognized as the *r*NTD-S protein (false positive); thus, the final specificity of the test was decreased.

### 3.4. Seroepidemiology Analysis in Swine Sera Collection

**Figure 4:**
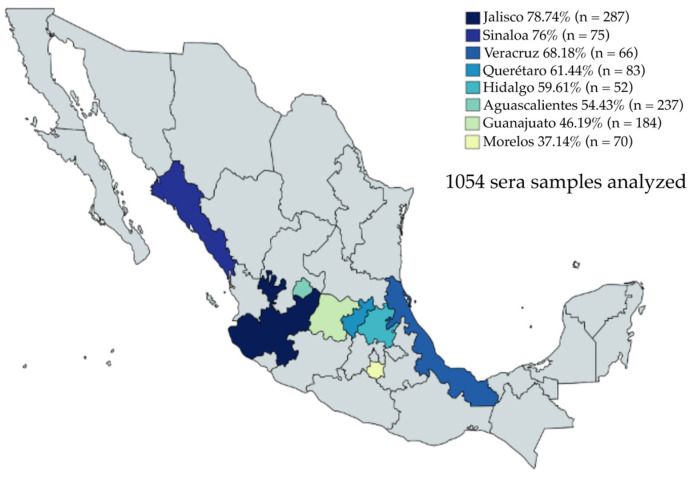
Percentage of positive sera samples for each evaluated state by iELISA. The highest percentage of positive sera was observed in Jalisco (*n* = 287) with 78.74%, and the lowest was observed in Morelos (*n* = 70) with 37.14%.

A collection of 1054 sera samples from eight Mexican states—Jalisco, Sinaloa, Veracruz, Querétaro, Hidalgo, Morelos, Aguascalientes, and Guanajuato—were evaluated by iELISA (Figure 4). The sera samples were collected from 2019 to 2021. Anti-PEDV IgG was detected in 650/1054 sera, representing a positivity rate of 61.66%. The seropositivity to PEDV was present in all evaluated states, with Jalisco showing the highest percentage of positive samples, with a 78.74% positivity rate (226/287) and the second-highest average OD_450nm_ value (0.611). Interestingly, Sinaloa, despite having a small number of positive serum samples, 57/75, represented 76% of the positive serum samples; furthermore, Sinaloa had the highest average OD_450nm_ value (0.794). Likewise, Veracruz, which also had a small number of serum samples, had a positivity rate of 68.18% (45/66), with an average OD_450nm_ value of 0.581. Querétaro had a 61.44% (51/83) sample positivity rate with an average OD_450nm_ value of 0.481. There were 52 sera samples evaluated from Hidalgo, 31 samples were positive, representing a positivity rate of 59.61%, and the average OD_450nm_ value was 0.606. Aguascalientes showed 54.43% positive samples (129/237) and the average OD_450nm_ value was 0.439. Guanajuato had 46.19% positive samples (85/184) and the average OD_450nm_ value was one of the lowest at 0.436. Finally, a striking result was obtained in the samples from Morelos, where 26 of 70 serum samples were positive, representing 37.14% seropositivity, and the lowest average OD_450nm_ value of 0.331, which was even below the cut-off. This result indicates a close relationship between the percentage of positive samples and the average OD_450nm_ value.

**Figure 5:**
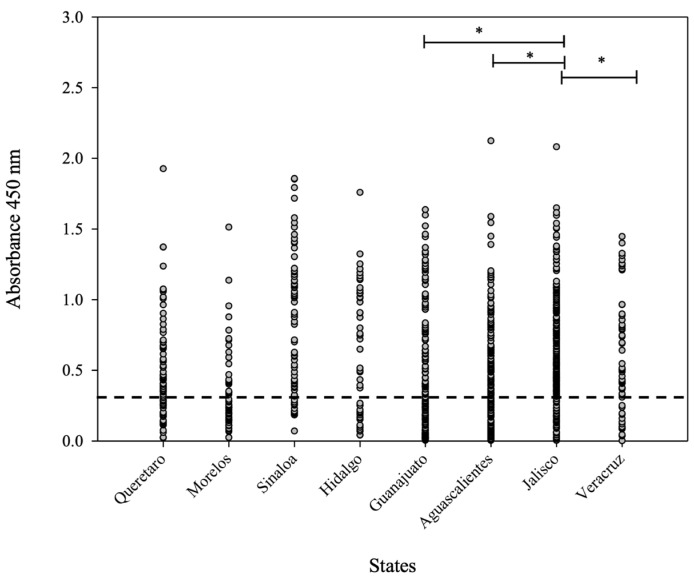
OD_450nm_ average value distribution for the 1054 sera analyzed by iELISA. The dashed line indicates the cut-off value. One-way ANOVA was used to compare the average value of OD_450nm_ considering the main pig-producing states (Aguascalientes, Jalisco, and Veracruz). Differences at *p* < 0.05 were considered statistically significant with a 95% confidential interval (* *p* < 0.05).

Statistical analysis carried out using one-way ANOVA showed a significant difference (* *p* < 0.05) between the evaluated sera from the main pig-producers states: Jalisco and Veracruz (Figure 5). It is important to mention that Jalisco has a high number of positive sera and more positive samples than Veracruz. Furthermore, between Jalisco and Guanajuato, there is a significant difference (* *p* < 0.05) in relation to the average OD_450nm_ value, even though the “*n*” values for both states were similar. Finally, Aguascalientes is a state ubicated near Jalisco (with a similar “*n*” number); however, there was a significant difference (* *p* < 0.05), since the average OD_450nm_ values were different in both states.

**Figure 6:**
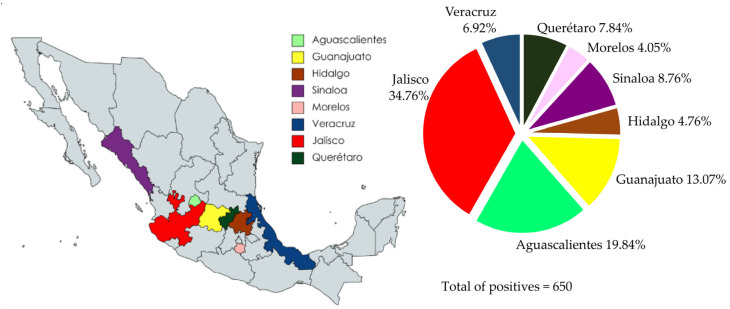
Distribution of overall positive serum samples evaluated using iELISA. The distribution of positive sera showed that Jalisco has a higher percentage of positive sera (226/650), 34.76%.

The percentage of positive serum samples was also higher than the average OD_450nm_ value. The local distribution of the serological results is summarized in Figure 6. Jalisco shows the highest number of positive samples with 226 (226/650), representing 34.76% of the positive serum samples; moreover, the sera of this state had a higher average OD_450nm_ value. Aguascalientes shows 129/650 (19.84%) positive serum samples; however, the “n” of the samples was higher than Jalisco (237). Therefore, Aguascalientes is in sixth place in terms of the percentage of positive individuals. From the Guanajuato sera samples, 85/650 (13.07%) were positive; however, this state shows a lower number of positive sera and lower average OD_450nm_ values. Sinaloa, Querétaro, and Veracruz show a similar number of positive serum samples, with 57 (8.76%), 51 (7.84%), and 45 (6.92%), respectively. Nonetheless, Sinaloa had the highest average OD_450nm_ value and a low “*n*”. Finally, Hidalgo and Morelos were the states with the lowest number of positive sera, 31/650 and 26/650, with 4.76% and 4%, respectively.

## 4. Discussion

The World Organization for Animal Health (WOAH) officially recognized the first outbreak of PED in Mexico in 2014, which caused significant economic losses in the swine industry. However, recent studies on PEDV in Mexico have focused on the molecular characterization of structural genes [12,22,25]. In Mexico, no seroepidemiology studies have been conducted; therefore, our work presents the first seroepidemiology study on 1054 sera samples using an iELISA with an *r*NTD of the S protein (*r*NTD-S) as the antigen. This study agrees with Myint et al., 2019 [26] and Lin et al., 2018 [19], where the NTD-S region was selected as the antigen due to the high antigenicity level compared to other structural proteins, producing persistent anti-S antibodies for a long time [27,28]. On the other hand, the selection of the NTD fragment is due to the presence of several conserved epitopes [26] that can induce the production of antibodies despite the genogroup. Furthermore, producing recombinant protein from an NTD segment (~45 kDa) of the S protein is more efficient in terms of expression simplicity, solubility, and yield than using a complete S-PEDV (180–220 kDa) protein with high molecular weight. Therefore, a protein fragment, usually >60 kDa, decreases the yield and solubility of a recombinant protein [29]. Generally, proteins with low molecular weight tend to produce soluble and functional forms of recombinant proteins. This is partly due to the requirement for fewer folding intermediates in the protein-folding pathway [30].

Thus, the NTD-S region was selected as the antigen to evaluate seropositive pigs. The iELISA developed in this study presented a high degree of sensitivity and specificity, allowing for the evaluation of many samples with both high sensitivity and specificity. The quantification of pigs who have been exposed to infection is ideally performed by direct testing for the presence of PEDV (prevalence); however, identifying cases in this way is dependent on capturing those cases while a pig is freeing the infection. On the other hand, estimating the number of pigs who have previously been infected with PEDV (seroepidemiology) enables an understanding of how the virus spreads in various locations, to retrospectively evaluate the performance of infectious disease, and hence plan for further outbreaks. The recombinant NTD-S region has been widely used to develop different immunoassays [17]. Therefore, the *r*NTD-S produced in this study allowed confirming positive and negative sera by Western blotting and the use of them as control sera during the iELISA standardization. Consequently, the optimal conditions of the iELISA allowed us to successfully evaluate the sera samples from different pig farms from different regions of Mexico. In this study, the determined cut-off value of 0.335 coincides with the values obtained by Myint et al., 2019 [26], and Lin et al., 2018 [19] using the NTD-S protein region, 0.320 and 0.286, respectively; thus, the cut-off value is appropriate and can be used to discriminate between positive and negative sera samples. ELISAs based on recombinant PEDV structural proteins increase specificity but sensitivity can be reduced due to the heterogenicity of PEDV isolates [19,26]; however, in this study, the percentages of sensitivity and specificity were 96.77% and 100%, respectively, and the concordance Κ (0.966) was not affected using the *r*NTD-S in the iELISA as an antigen. These percentages of sensitivity and specificity are similar to those obtained by Myint et al., 2019 (92.6 and 90.1%, respectively) [26], and Lin et al., 2018 (96.71 and 96.77%, respectively) [19]. Statistically, the sum of the sensitivity and specificity values for a test to be useful should be at least 1.5 (<1, which is a useless test, and 2, which is a perfect test) [31]. In our study, we obtained 0.9677 for sensitivity and 1.0 for specificity, which gives 1.9677, suggesting an acceptable statistical value support for the iELISA test developed in this study.

Data from the Fideicomisos Instituidos en Relación con la Agricultura (FIRA), obtained in 2020 [32], showed that Jalisco, Sonora, Puebla, Guanajuato, Veracruz, and Yucatan are the states with the highest pork production in Mexico. Together, these states register 47.8% of the total pig population and 60.5% of pork meat production; http://infosiap.siap.gob.mx/repoAvance_siap_gb/pecAvanceProd.jsp (accessed on 20 June 2023). In this study, sera samples from three of these key swine-producing states (Jalisco, Guanajuato, and Veracruz) were analyzed, suggesting a direct relationship between the seroprevalence of PEDV and the high capacity of production. Recently, Castañeda-Montes et al., 2023 [33] analyzed sera samples by an iELISA with a recombinant Porcine Deltacoronavirus (PDCoV) membrane protein. This study was conducted on sera from the central region of Mexico, which includes Jalisco and Guanajuato. The results showed that PDCoV was still circulating in the region, emphasizing the need to examine the presence of swine coronavirus, coinfections, and its impact on pig farming in Mexico.

## 5. Conclusions

Our seroepidemiological study performed via iELISA with *r*NTD-S suggests that this protein is an excellent antigen with potential use in diagnostic systems. PEDV still circulates in the main swine producers’ regions of Mexico; therefore, the impact of PEDV on national swine production may be greater than expected. Seroepidemiology studies are necessary for the prevention and control of the disease. Therefore, the iELISA method used in this study can be used for the seroepidemiological evaluation of PEDV in the swine industry.

## Figures and Tables

**Table 1:** The 2 × 2 Contingency table.

	PED Positive	PED Negative	Total
Positive serum	*a*	*b*	*r = a + b*
Negative serum	*c*	*d*	*s = c + d*
Total	*t = a + c*	*u = b + d*	*N = a + b + c +d*

**Table 2:** Summary of the origin of sera samples.

State	Sera Samples	Sera Origin
Aguascalientes	237	Slaughterhouse
Guanajuato	184	Slaughterhouse
Hidalgo	52	Backyard production
Jalisco	287	Slaughterhouse
Morelos	70	Backyard production
Querétaro	83	Backyard production
Sinaloa	75	Backyard production
Veracruz	66	Slaughterhouse
Total	1054	

## Data Availability

Not applicable.

## Supplementary Materials

The following supporting information can be downloaded at: https://www.mdpi.com/article/10.3390/microorganisms11071843/s1, Table S1: Data collection of iELISA assay from all serum samples obtained from farms.
